# Pertuzumab in Combination with Trastuzumab and Docetaxel as Adjuvant Doublet Therapy for HER2-Positive Breast Cancer: A Systematic Review

**DOI:** 10.3390/ijms26051908

**Published:** 2025-02-23

**Authors:** Ignacio Ventura, Nerea Pinilla Salcedo, Marcelino Pérez-Bermejo, Javier Pérez-Murillo, Manuel Tejeda-Adell, Francisco Tomás-Aguirre, María Ester Legidos-García, María Teresa Murillo-Llorente

**Affiliations:** 1 Molecular and Mitochondrial Medicine Research Group, School of Medicine and Health Sciences, Catholic University of Valencia San Vicente Mártir, C/Quevedo nº 2, 46001 Valencia, Spain; ignacio.ventura@ucv.es; 2 Translational Research Center San Alberto Magno CITSAM, Catholic University of Valencia San Vicente Mártir, C/Quevedo nº 2, 46001 Valencia, Spain; 3 School of Medicine and Health Sciences, Catholic University of Valencia San Vicente Mártir, C/Quevedo nº 2, 46001 Valencia, Spain; nepisal@mail.ucv.es; 4 SONEV Research Group, Faculty of Medicine and Health Sciences, Catholic University of Valencia San Vicente Mártir, C/Quevedo nº 2, 46001 Valencia, Spain; javier.perezmu@ucv.es (J.P.-M.); manuel.tejeda@ucv.es (M.T.-A.); paco.tomas@ucv.es (F.T.-A.); ester.legidos@ucv.es (M.E.L.-G.); mt.murillo@ucv.es (M.T.M.-L.)

**Keywords:** breast cancer, human epidermal growth factor receptor 2 (HER2), trastuzumab, pertuzumab

## Abstract

Breast cancer is the most clinically relevant pathology of the mammary gland and is currently the most diagnosed malignant disease among women worldwide. In breast cancer prevention, it is important to consider that the risk of developing the disease is not the same for the entire population. This pathology presents heterogeneous clinical manifestations and the most common classification is related to the following hormonal receptors: estrogen receptor (ER), progesterone receptor (PR), human epidermal growth factor receptor 2 (HER2), and triple-negative (TNBC). Currently, a new class of therapy is being used for cancer treatment: anti-body-drug conjugates. A bibliographic search was performed by establishing keywords and then combining them using Boolean operators OR and AND. Thus, the search equation was formulated according to the PICO search question to be used in the PubMed database. Results: Fifteen studies that met the established inclusion criteria were analyzed, and their methodological quality was assessed using the Joanna Briggs Institute approach, demonstrating high reliability in the results obtained, the analyzed studies focus on the combination of adjuvant Pertuzumab + Trastuzumab with chemotherapy for the treatment of HER2-positive breast cancer. Scientific evidence suggests that the combination of pertuzumab and trastuzumab not only improves the survival of patients with HER2-positive breast cancer but also provides a safe and flexible treatment option.

## 1. Introduction

Breast cancer (BC) is the most clinically relevant pathology of the mammary gland. It has become the most commonly diagnosed malignancy in women worldwide and is the leading cause of cancer death in women aged 20–59 years according to the latest GLOBOCAN data. However, it is rare in men, accounting for 1% of cancer diagnoses. BC (Breast Cancer) is a disease with heterogeneous clinical manifestations and is classified into numerous subtypes that have evolved over the years [1,2]. The most common and widely accepted classification is based on an immunohistochemical perspective related to the following hormone receptors: estrogen receptor (ER), progesterone receptor (PR), and human epidermal growth factor (HER2). It is also considered an important marker in the presence and recurrence of tumors and triple-negative (TNBC) [3]. The following subtypes of breast cancer are identified: Luminal A, Luminal B, HER2 positive, and triple negative.

Breast cancer staging is used to classify patients into risk categories that determine prognosis and treatment recommendations. It is based on physical examination and imaging studies, as well as pathological examination of the primary tumor and lymph nodes. The most commonly used TNM staging system is that of the American Joint Committee on Cancer, which divides patients into four groups according to the size of the primary tumor (T), regional lymph node status (N), and the presence of distant metastases (M) [4]. Stage 0 includes non-invasive BC and ductal carcinoma in situ. Stages I, IIa, and IIb represent early invasive BC. Stages IIIa, IIIb, and IIIc include locally advanced BC, while stage IV includes all metastatic BC. Stage II breast cancer is divided into stages IIa and IIb based on the size of the tumor and the involvement of lymph nodes. Stage IIa refers to tumors that are either smaller than 2 cm but have spread to 1–3 axillary lymph nodes or are 2–5 cm in size without lymph node involvement. In contrast, stage IIb describes tumors that are 2–5 cm in size with 1–3 lymph nodes affected or larger than 5 cm without nodal involvement. Stage III breast cancer, categorized into IIIa, IIIb, and IIIc, reflects more advanced disease. Stage IIIa involves extensive lymph node involvement (4–9 axillary nodes or internal mammary nodes) regardless of tumor size, while stage IIIb includes tumors of any size that have invaded the chest wall or skin. Stage IIIc is defined by the involvement of 10 or more axillary lymph nodes, infraclavicular or supraclavicular nodes, or both, regardless of tumor size or invasion of nearby structures [4,5].

Breast cancer has become the most commonly diagnosed cancer worldwide, surpassing lung and prostate cancers. Developed countries have the highest incidence of breast cancer, although this may be due to better detection of this pathology. The mortality rate varies widely among regions, being higher in low socioeconomic areas due to fewer resources to detect the disease and difficulty in accessing adequate treatment [6].

In women’s health screening, it is important to identify risk factors associated with a higher incidence of breast cancer. There are non-modifiable risk factors and modifiable risk factors. Non-modifiable risk factors are age, gender, histologic risk factors, genetic risk factors and family history, exogenous hormones, and reproductive risk factors. The modifiable risk factors are physical activity and diet, obesity, alcohol consumption, and smoking [4,7,8].

Treatments for BC have been extensively developed over the past few decades, and thanks to improved therapeutic approaches, most patients with early-stage breast cancer are curable. Although survival has improved for patients with advanced BC, it is still incurable. A new class of targeted cancer therapy is currently being used: antibody–drug conjugates (ADCs), which consist of a monoclonal antibody conjugated to a cytotoxic agent via a chemical linker. This unique structure develops specific cytotoxicity against tumor cells [2].

The standard first-line treatment for HER2-positive breast cancer has historically been trastuzumab in combination with chemotherapy. However, recent research suggests that the combination of pertuzumab and trastuzumab in the adjuvant setting may offer significant benefits in terms of survival and response to treatment. This study is justified by the need to determine the feasibility and efficacy of combination therapy with pertuzumab and trastuzmab in critically ill patients.

**Figure 1:**
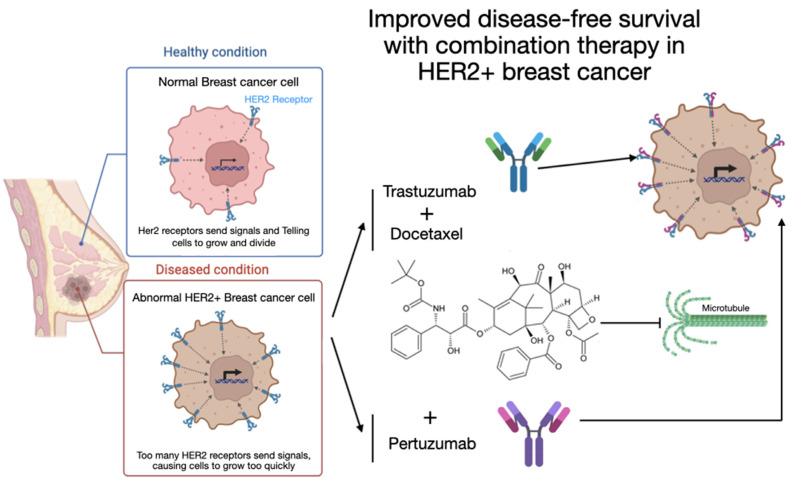
Improved disease-free survival with combination therapy in HER2+ breast cancer: This illustrative schematic shows the difference between a healthy and a diseased (HER2+) breast cancer cell. In the healthy state, HER2 receptors on the cell surface send signals that regulate cell growth and division. In the diseased state, an excess of HER2 receptors sends more signals than normal, leading to uncontrolled cell growth. Combination therapy with trastuzumab, docetaxel, and pertuzumab targets these HER2 receptors and improves disease-free survival in patients with HER2+ breast cancer. Trastuzumab and pertuzumab are monoclonal antibodies that target the HER2 receptor at different sites. Trastuzumab blocks cell proliferation by inhibiting receptor dimerization, while pertuzumab prevents growth signals by binding to a different HER2 epitope. Docetaxel prevents the polymerization of microtubules necessary for tumor cell mitosis, thereby interrupting cell division and promoting cell death.

It is critical to evaluate the advisability and safety of this therapeutic combination in the ICU setting, where patients may have increased frailty and associated complications. In addition, it is important to analyze the potential side effects and their management in the ICU setting to ensure that the implementation of this combination therapy does not compromise patient stability. Improving disease-free survival with combination therapy in HER2+ breast cancer: This illustrative schematic shows the difference between a breast cancer cell in the healthy state and one in the diseased (HER2+) state. In the healthy state, HER2 receptors on the cell surface send signals that regulate cell growth and division. In the diseased state, an excess of HER2 receptors sends more signals than normal, leading to uncontrolled cell growth. Combination therapy with trastuzumab, docetaxel, and pertuzumab targets these HER2 receptors and improves disease-free survival in patients with HER2+ breast cancer. Trastuzumab and pertuzumab are monoclonal antibodies that target the HER2 receptor at different sites. Trastuzumab blocks cell proliferation by inhibiting receptor dimerization, while pertuzumab prevents growth signaling by binding to a different HER2 epitope. Docetaxel is an antimitotic agent that inhibits microtubule depolymerization by specifically binding to tubulin subunits, thereby stabilizing microtubule assembly and disrupting cell division (Figure 1). Given the diverse clinical scenarios in HER2-positive breast cancer, including neoadjuvant, adjuvant, and metastatic settings, this review aims to systematically evaluate the efficacy and safety of combining pertuzumab, trastuzumab, and docetaxel. To address the heterogeneity of patient populations and study designs, we categorized the evidence based on treatment intent, end points, and toxicity profiles. This approach seeks to provide a comprehensive yet structured analysis, facilitating the translation of findings into clinical practice.

## 2. Methods

This work is a systematic review conducted in accordance with the criteria set out in the Preferred Reporting Items for Systematic Reviews and Meta-Analysis (PRISMA) [9] guidelines which was registered in the records of the Open Science Framework (https://osf.io/gsyme; accessed 23 December 2024). The PRISMA 2020 checklist (Supplementary Data S1) was also applied. The main idea is to know the use of the biopharmaceutical trastuzumab in combination with pertuzumab in the treatment of breast cancer, as well as its adverse effects. The search was conducted in the Cochrane and PubMed databases to ensure a broad and rigorous coverage of the available scientific literature. The equation was formed by combining the previously established descriptors using the Boolean operators “AND” and “OR” and is as follows ((HUMAN EPIDERMAL GROWTH FACTOR) OR (HER2)) AND (BREAST CANCER OR BREAST CARCINOMA OR BREAST NEOPLASIA) AND PERTUZUMAB AND TRASTUZUMAB AND CHEMOTHERAPY.

To conduct this literature search, it is necessary to formulate the research question following the steps of the PICO format (Table 1). The question is as follows: “In patients with HER2-positive breast cancer, is the combined treatment of pertuzumab and trastuzumab more effective than trastuzumab and conventional chemotherapy in improving progression-free survival and pathologic complete response in controlled clinical trials?

### 2.1. Inclusion and Exclusion Criteria

#### 2.1.1. Inclusion Criteria

- Published studies with full text: only studies that are available in their entirety will be considered to ensure a comprehensive and accurate analysis.
- Human studies: the search will focus exclusively on studies with human patients to ensure the clinical relevance of the results.
- Published within the last 10 years: to ensure that the data are current and reflect the most recent advances in the treatment of HER2-positive breast cancer.
- Published in English: English language studies will be selected to ensure accessibility and consistency in the literature review.

#### 2.1.2. Exclusion Criteria

- Animal studies: studies using animal models will be excluded as the results may not be directly applicable to humans.
- Studies without scientific evidence: studies that do not present data based on sound scientific evidence or that do not meet adequate methodological standards will be discarded.

**Figure 2:**
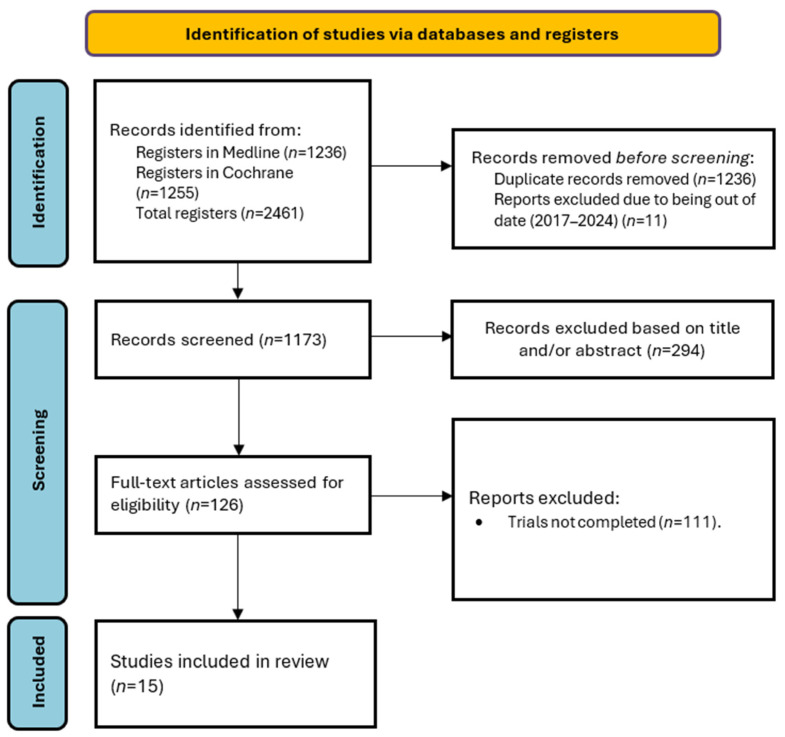
PRISMA flowchart of the study selection process.

The initial search yielded a total of 1236 articles. The flowchart in Figure 2 describes the screening and selection process. 

## 3. Results and Discussion

After a first reading of the title and results, the selection was reduced to 1173 articles, of which 40 were excluded after a complete reading of each and after verifying that they were not the objective of this work. Applying the inclusion and exclusion criteria, 126 articles were selected, and after the number of records excluded due to incompatibility with the purpose of the study, 15 articles were included. These studies were graded according to the corresponding checklist of the Joanna Briggs Institute (JBI) [10] and all of them were scored as 100%. Table 2 shows the main characteristics of each study.

In the treatment of HER2-positive breast cancer, recent research has demonstrated the efficacy of therapeutic combinations including pertuzumab and trastuzumab in various clinical settings, as well as a favorable safety profile in various populations. The phase III KRISTINE trial compared neoadjuvant treatment with trastuzumab emtasine and pertuzumab with a combination of docetaxel, carboplatin, trastuzumab, and pertuzumab. The results showed a complete pathologic response rate of 44.4% in the first group and 55.7% in the second, with adverse events including plateletopenia and fatigue in the em-tasine group and neutropenia and diarrhea in the trastuzumab and pertuzumab chemo-therapy group, with these effects being more frequent in the latter group [14].

In the PERTAIN trial, patients with HER2-positive, hormone-receptor-positive metastatic breast cancer treated with pertuzumab, trastuzumab, and an aromatase inhibitor showed a median progression-free survival of 18.89 months compared to 15.80 months with trastuzumab alone. The combination therapy demonstrated a safety profile consistent with previous studies, with the most common adverse events being diarrhea, alopecia, and nausea, while left ventricular ejection fraction remained stable in most patients [15].

The DESTINY-Breast03 trial demonstrated that trastuzumab deruxtecan achieved a median progression-free survival of 29.1 months, outperforming trastuzumab emtasine at 7.2 months. In addition, trastuzumab deruxtecan showed a significant benefit in overall survival, although adverse events such as pneumonitis and pneumonia necessitated the discontinuation of treatment in some cases [16].

The PUFFIN study, conducted in Chinese patients with HER2-positive breast cancer, reported a median progression-free survival of 16.5 months with Pertuzumab compared to 12.5 months in the placebo group. Safety and efficacy results were consistent with the CLEOPATRA study, with leukopenia being the most common adverse event [17]. On the other hand, the MARIANNE trial evaluated trastuzumab emtasine, both as monotherapy and in combination with pertuzumab, versus trastuzumab plus taxane in patients with HER2-positive advanced breast cancer. The results showed that trastuzumab emtasine and its combination with pertuzumab were not superior to trastuzumab and taxane in terms of progression-free survival, although they had a lower incidence of serious adverse events such as alopecia and diarrhea compared to the control treatment [18].

The Chinese subgroup of the APHINITY trial evaluated the adjuvant use of pertuzumab in combination with trastuzumab and standard chemotherapy and demonstrated greater efficacy and a safety profile consistent with the overall population. The most common adverse events were diarrhea and febrile neutropenia, and no serious cardiac events were observed in this group [19]. In the Neo-PATH trial, which evaluated the combination of atezolizumab, docetaxel, trastuzumab, and pertuzumab in patients with stage II or III HER2-positive breast cancer, a pathologic complete response rate of 61% was achieved, with a higher response rate in hormone receptor-negative (77%) compared to hormone receptor-positive (44%) patients. The most common adverse events were febrile neutropenia and encephalitis [20].

The PEONY study reported a pathologic complete response rate of 39.3% in the per-tuzumab group versus 21.8% in the placebo group, showing a significant improvement with treatment. Adverse events such as neutropenia were more common in the pertuzumab group [21]. In addition, the PERUSE trial, which included patients with HER2-positive, hormone-receptor-positive breast cancer, showed a median progression-free survival of 20.7 months and overall survival of more than five years in this group, supporting the safety and efficacy of the combination of pertuzumab, trastuzumab, and a tax-ane, consistent with the results of the CLEOPATRA trial [22].

In a prospective multinational trial evaluating the combination of pertuzumab and trastuzumab with adjuvant chemotherapy, an improvement in 3-year disease-free survival was observed in patients with HER2-positive breast cancer, especially in those with positive nodes, although no significant differences were found in node-negative patients. Diarrhea was the most common adverse event in the pertuzumab group [23]. Finally, the MetaPHER trial confirmed the efficacy of subcutaneous trastuzumab in combination with pertuzumab and docetaxel in patients with HER2-positive breast cancer, with a progres-sion-free survival of 18.7 months and adverse events such as neutropenia and hypertension, suggesting greater flexibility in patient management [24].

Taken together, these studies support the use of pertuzumab and trastuzumab in combination to improve progression-free survival and overall survival in patients with HER2-positive breast cancer, while maintaining a favorable safety profile compared to standard of care. The following is a summary of the major clinical trials that evaluated combination therapies in patients with HER2-positive breast cancer, highlighting the efficacy and most common adverse events associated with the therapies. The table summarizes key data from each trial, including progression-free survival, pathologic complete response, overall survival, and the most common adverse events observed in the different treatment groups. These studies provide evidence on the efficacy of various therapeutic combinations, such as trastuzumab, pertuzumab, and trastuzumab deruxtecan, compared to standard of care and placebo, and help guide clinical decisions in the treatment of HER2-positive breast cancer. The results are presented in separate sections for neoadjuvant, adjuvant, and metastatic settings to ensure a clear understanding of the efficacy and safety of pertuzumab-based regimens within each clinical context (Table 3).

The heterogeneity in study populations and endpoints posed challenges for direct comparisons. However, by stratifying the evidence and focusing on consistent measures such as progression-free survival and toxicity profiles, we provide a comprehensive synthesis that informs clinical decision-making. HER2-positive breast cancer (HER2+ BC) has become one of the leading causes of morbidity and mortality in women worldwide, particularly those between the ages of 20 and 59. This form of cancer represents a clinically relevant subtype of breast pathology characterized by overexpression of the HER2 receptor and its involvement in tumor aggressiveness. Historically, the first-line treatment for HER2+ BC has been chemotherapy; however, the development of targeted therapies has significantly changed the treatment approach in recent years, allowing the integration of drugs such as trastuzumab and pertuzumab with chemotherapeutic agents such as docetaxel. This systematic review is based on a comprehensive analysis of recent studies and examines the benefits and potential adverse effects of this therapeutic combination compared with conventional options.

In the 2020 CLEOPATRA study, a randomized phase III trial, Swain et al. investigated the combination of pertuzumab, Trastuzumab, and Docetaxel compared to a treatment without pertuzumab. The findings showed a significant improvement in progression-free survival and overall survival in the group treated with the triple combination [11]. 

These results are consistent with those reported by Nicholas J. Rober et al. [12], who approved the use of pertuzumab in combination with Trastuzumab and Docetaxel in patients without prior chemotherapy, observing a median progression-free survival of 16.9 months. In addition, the study by Vázquez et al. [13] compared Paclitaxel dual therapy with dual therapy versus Paclitaxel plus Trastuzumab, again finding a significant improvement in survival and clinical tumor response.

The KRISTINE trial by Hurvitz et al. [14], which included pertuzumab, trastuzumab, and docetaxel along with carboplatin, also demonstrated an increase in pathologic response. In a more recent study, DESTINY-Breast03, the same author demonstrated that trastuzumab deruxtecan showed a progression-free survival of 29.1 months compared to 7.2 months achieved with trastuzumab emtasine [16]. For its part, the PERTAIN study by Mothaffar Rimawi et al. [16] reaffirmed the safety of the dual therapy and reported a median survival of 18.89 months in the pertuzumab group versus 15.80 months in the group treated with trastuzumab alone. The PUFFIN trial conducted in China by Binghe Xu et al. [17] showed results consistent with the CLEOPATRA trial in terms of the efficacy of triple therapy, consolidating the benefit of pertuzumab in combination with trastuzumab and docetaxel in terms of risk-benefit.

Additional studies, such as MARIANNE [18] by Edith Perez et al. which included a taxane, and PEONY by Zhimin Shao et al. [21] support these findings, highlighting an improvement in complete pathologic response in the group treated with pertuzumab, trastuzumab, and docetaxel. Similarly, the subset of the APHINITY trial conducted by Zhimin Shao et al. [19,20] highlighted the efficacy of adding pertuzumab as adjuvant therapy after surgery in patients with HER2+ early CM [19]. In smaller studies, such as the phase II study by Ahn et al. [20], the combination of pertuzumab, trastuzumab, docetaxel, and atezolizumab reported a pathologic complete response rate of 61%.

At the safety level, adverse effects (AEs) associated with pertuzumab in combination with trastuzumab and chemotherapy include symptoms such as diarrhea, fatigue, nausea, and peripheral neutropenia, as observed in the study by Nicholas J. Rober et al. [12]. Recent studies such as PUFFIN (2022) and PERTAIN (2018) also identified diarrhea, alopecia, and leukopenia as the most common adverse events [15,17]. Regarding cardiac function, several studies, including MetaPHER by Kuemmel et al. [23] and the study by Minckwitz et al. [24], reported a stable left ventricular ejection fraction (LVEF), albeit with a higher incidence of diarrhea and other AEs in the pertuzumab group.

Other studies include the PERUSE trial by Millas et al. [22], which confirmed the safety and tolerability of combination therapy, and the retrospective study by Young Pio Lee et al. [25], which reported an overall response rate of 86.8% in patients with previously un-treated metastatic disease. Subcutaneous administration of trastuzumab, as evaluated by Kuemmel et al. in MetaPHER, was found to be an effective and safe option, providing additional flexibility for patients [24]. In conclusion, current evidence suggests that the combination of pertuzumab, trastuzumab, and chemotherapy in the treatment of HER2+ CM offers clear benefits in terms of survival and clinical response. However, the adverse event profile, particularly neutropenia and diarrhea, requires appropriate monitoring to optimize patient safety and treatment efficacy. These findings re-emphasize the potential of combination regimens as a first-line option in HER2+ CM and suggest their inclusion in clinical guidelines, particularly for patients at high risk of relapse.

This review makes a unique contribution by systematically evaluating the efficacy and safety of the combination of pertuzumab, trastuzumab, and docetaxel in different clinical settings. In contrast to previous reviews, this study integrates results from recent clinical trials and highlights the differences in treatment response in different patient populations. By providing a structured comparison of key studies, this review advances the current understanding of dual HER2 blockade and its role in optimizing treatment strategies for HER2-positive breast cancer.

This review highlights the potential impact of combining pertuzumab and trastuzumab with chemotherapy on future breast cancer research. By synthesizing data from multiple clinical trials, this analysis identifies gaps in current knowledge and underscores the need for further investigation to optimize treatment regimens, understand long-term safety profiles, and explore novel therapeutic combinations. Future studies should focus on refining patient selection criteria and evaluating real-world outcomes to ensure that this therapeutic approach benefits a broader range of patients with HER2-positive breast cancer.

## 4. Conclusions

This systematic review highlights the clinical efficacy and safety of combining pertuzumab, trastuzumab, and docetaxel across different treatment settings for HER2-positive breast cancer, including neoadjuvant, adjuvant, and metastatic contexts. While the heterogeneity of patient populations, endpoints, and study designs poses challenges for direct comparisons, this diversity also reflects the versatility of this combination therapy in addressing various clinical needs. To provide clearer insights, we categorized the included studies based on treatment intent (neoadjuvant, adjuvant, or metastatic), outcome measures (progression-free survival, overall survival, or pathologic complete response), and toxicity profiles. This structured approach allows for a more nuanced understanding of the therapeutic potential and limitations of pertuzumab-based regimens, emphasizing their role as a cornerstone in the treatment of HER2-positive breast cancer. Advances in the treatment of HER2-positive breast cancer underscore the efficacy of the combination of pertuzumab, trastuzumab, and docetaxel, which has demonstrated a significant improvement in disease-free survival over conventional treatment. However, this combination therapy is not without side effects, with neutropenia and diarrhea being the most common adverse events. Careful consideration of these risks by healthcare professionals is essential to optimize treatment planning and follow-up. In addition, adjuvant dual therapy with pertuzumab and trastuzumab appears to be a promising first-line option, especially for patients at high risk of recurrence, suggesting an important step towards more effective and personalized treatments in oncology. Future studies should focus on standardizing endpoints and treatment protocols across settings to further refine the role of pertuzumab in HER2-positive breast cancer management.

## Figures and Tables

**Table 1:** PICOS research question.

Patient (P)	HER2-positive breast cancer patients.
Intervention (I)	Comparison of the binding of two drugs to HER2+ breast cancer progression.
Comparison (C)	Pertuzumab combined with trastuzumab versus trastuzumab with conventional chemotherapy.
Outcome (O)	Efficacy of combination therapy and improvement in progression-free survival and pathologic complete response rate.

**Table 2:** The main characteristics of each study were analyzed.

Ref.	Year	n	Objective	Intervention	Main Outcomes	Most Common AE
[11]	2020	808	Compare the efficacy and safety of Pertuzumab + Trastuzumab + Docetaxel vs. Placebo + Trastuzumab + Docetaxel	Pertuzumab + Trastuzumab + Docetaxel vs. Placebo + Trastuzumab + Docetaxel	Improved median overall survival (57.1 months vs. 40.8 months)	Neutropenia Congestive heart failure
[12]	2017	266	Evaluate treatment patterns and outcomes in real-world settings	Trastuzumab + Pertuzumab + Taxane	Median progression-free survival: 16.9 months	Fatigue Diarrhea Nausea Peripheral neuropathy
[13]	2023	2836	Assess the benefits of dual HER2 therapy over single HER2 therapy	Dual HER2 therapy vs. Single HER2 therapy	Increased complete pathologic response	Varied across studies
[14]	2018	444	Compare neoadjuvant regimens for HER2+ BC	Trastuzumab emtansine + Pertuzumab vs. Docetaxel + Carboplatin + Trastuzumab + Pertuzumab	Higher complete pathologic response with chemotherapy combination	Neutropenia Diarrhea
[15]	2018	258	Evaluate the impact of adding Pertuzumab to Trastuzumab + Aromatase Inhibitor	Pertuzumab + Trastuzumab + Aromatase Inhibitor vs. Trastuzumab alone	Median progression-free survival: 18.89 vs. 15.80 months	Diarrhea Alopecia Nausea
[16]	2023	524	Compare Trastuzumab deruxtecan vs. Trastuzumab emtasine	Trastuzumab deruxtecan vs. Trastuzumab emtasine	PFS: 29.1 months vs. 7.2 months	Pneumonitis, Pneumonia
[17]	2023	243	Evaluate Pertuzumab in combination therapy	Pertuzumab + Trastuzumab + Docetaxel vs. Placebo	Median PFS: 16.5 vs. 12.5 months	Leukopenia Anemia Diarrhea
[18]	2017	1095	Compare Trastuzumab emtansine ± Pertuzumab vs. Trastuzumab + Taxane	Trastuzumab emtansine ± Pertuzumab vs. Trastuzumab + Taxane	No superiority in PFS	Lower alopecia and diarrhea in the emtansine arm
[19]	2021	558	Evaluate adjuvant therapy with Pertuzumab + Trastuzumab	Pertuzumab + Trastuzumab + Chemotherapy	Improved adjuvant treatment outcomes	Diarrhea Febrile neutropenia
[20]	2022	67	Assess neoadjuvant therapy efficacy	Atezolizumab + Docetaxel + Trastuzumab + Pertuzumab	Pathologic complete response: 61%	Febrile neutropenia Encephalitis
[21]	2020	329	Compare Pertuzumab vs. placebo in early BC	Pertuzumab + Trastuzumab + Docetaxel vs. Placebo	Higher pathologic response in the Pertuzumab group	Neutropenia
[22]	2021	1436	Assess the safety and tolerability of combination therapy	Pertuzumab + Trastuzumab + Taxane	Median PFS: 20.7 months	Neutropenia Diarrhea
[23]	2017	4085	Evaluate disease-free survival in an adjuvant setting	Pertuzumab + Trastuzumab + Chemotherapy vs. Placebo	Improved 3-year disease-free survival	Diarrhea
[24]	2021	276	Assess the safety of subcutaneous Trastuzumab	Subcutaneous Trastuzumab + Pertuzumab + Docetaxel	Median PFS: 18.7 months	Neutropenia Hypertension
[25]	2022	268	Analyze the real-world efficacy of Pertuzumab combinations	Various combinations including Pertuzumab	Median OS: 19.1 months	Variable, consistent with CLEOPATRA

**Table 3:** Summary of clinical trials evaluating the combination of pertuzumab, trastuzumab, and other therapies in HER2-positive breast cancer.

Study	Clinical Context	Treatment	PFS (Months)	PCR (%)	OS (Months)	Common AE
KRISTINE	HER2-positive breast cancer, neoadjuvant setting	Trastuzumab emtasina + Pertuzumab vs. Docetaxel + carboplatin + Trastuzumab + Pertuzumab	-	44.4% (emtasine) vs. 55.7% (chemotherapy)	-	Thrombocytopenia, fatigue (emtansine); neutropenia, diarrhea (chemotherapy
PERTAIN	HER2-positive metastatic breast cancer, hormone receptor-positive.	Pertuzumab + trastuzumab + aromatase inhibitor vs. trastuzumab alone	18.89 (com-bination) vs. 15.80 (Trastuzumab)	-	-	Diarrhea, alopecia, nausea; stable ejection fraction
DESTINY-Breast03	Advanced HER2-positive breast cancer	Trastuzumab deruxtecan vs. Trastuzumab emtasine	29.1 (derux-tecan) vs. 7.2 (emtasine)	-	Significant improvement in deruxtecan	Pneumonitis, neu-monia (treatment interrupted in some cases)
PUFFIN	HER2-positive breast cancer, Chinese patients	Pertuzumab vs. placebo	16.5 (pertuzumab) vs. 12.5 (placebo)	-	-	Leukopenia
MARIANNE	HER2-positive advanced breast cancer	Trastuzumab emtasine (alone or with Pertuzumab) vs. Trastuzumab + taxane	Not superior to Trastuzumab + taxane	-	-	Less alopecia and diarrhea in emtasin than in Trastuzumab + taxane.
APHINITY (China)	HER2-positive breast cancer, adjuvant use	Pertuzumab + Trastuzumab + standard chemotherapy	-	-	-	Diarrhea, febrile neutropenia; no serious cardiac events
Neo-PATH	HER2-positive breast cancer stages II-III	Atezolizumab + Docetaxel + Trastuzumab + Pertuzumab	-	61% (overall); 77% (negative HR) vs. 44% (positive HR)	-	Febrile neutropenia, encephalitis
PEONY	HER2-positive breast cancer	Pertuzumab vs. placebo	-	39.3% (Per-tuzumab) vs. 21.8% (placebo)	-	Neutropenia (frequent in the Pertuzumab group)
PERUSE	HER2-positive and hormone receptor-positive breast cancer	Pertuzumab + Trastuzumab + taxane	20.7	-	>60 months	Consistent safety and efficacy; CLEOPATRA-aligned results
Adjuvant assay	HER2-positive breast cancer, adjuvant use	Pertuzumab + Trastuzumab + chemotherapy	-	-	-	Diarrhea (frequent in the Pertuzumab group)
MetaPHER	HER2-positive breast cancer	Subcutaneous Trastuzumab + Pertuzumab + Docetaxel	18.7	-	-	Neutropenia, hypertension

PFS: progression-free survival; PCR: pathologic complete response; OS: overall survival.

## Data Availability

No new data were created or analyzed in this study.

## Supplementary Materials

The following supporting information can be downloaded at: https://www.mdpi.com/article/10.3390/ijms26051908/s1.

## Abbreviations

The following abbreviations are used in this manuscript:

ADC	Antibody–Drug Conjugate
BC	Breast Cancer
ER	Estrogen Receptor
HER2	Human Epidermal Growth Factor Receptor 2
OS	Overall Survival
PCR	Pathologic Complete Response
PFS	Progression-Free Survival
PR	Progesterone Receptor
TNBC	Triple Negative Breast Cancer

## References

[1] Aranda-Gutierrez A., Diaz-Perez H.M. (2024). Histology, Mammary Glands. 2023 May 1. StatPearls [Internet].

[2] Chen Y.F., Xu Y.Y., Shao Z.M., Yu K.D. (2023). Resistance to antibody-drug conjugates in breast cancer: Mechanisms and solutions. Cancer Commun..

[3] Orrantia-Borunda E., Anchondo-Nuñez P., Acuña-Aguilar L.E., Gómez-Valles F.O., Ramírez-Valdespino C.A., Mayrovitz H.N. (2022). Subtypes of Breast Cancer. Breast Cancer [Internet].

[4] Alkabban F.M., Ferguson T. (2024). Breast Cancer. [Updated 2022 Sep 26]. StatPearls [Internet].

[5] Trayes K.P., Cokenakes S.E.H. (2021). Breast Cancer Treatment. Am. Fam. Physician.

[6] Arzanova E., Mayrovitz H.N., Mayrovitz H.N. (2022). The Epidemiology of Breast Cancer. Breast Cancer [Internet].

[7] White M.C., Holman D.M., Boehm J.E., Peipins L.A., Grossman M., Henley S.J. (2014). Age and cancer risk: A potentially modifiable relationship. Am. J. Prev. Med..

[8] Admoun C., Mayrovitz H.N., Mayrovitz H.N. (2022). The Etiology of Breast Cancer. Breast Cancer [Internet].

[9] Liberati A., Altman D.G., Tetzlaff J., Mulrow C., Gøtzsche P.C., Ioannidis J.P., Clarke M., Devereaux P.J., Kleijnen J., Moher D. (2009). The PRISMA statement for reporting systematic reviews and meta-analyses of studies that evaluate healthcare interventions: Explanation and elaboration. BMJ.

[10] Jordan Z., Lockwood C., Munn Z., Aromataris E. (2019). The updated Joanna Briggs Institute Model of Evidence-Based Healthcare. Int. J. Evid. Based Healthc..

[11] Swain S.M., Miles D., Kim S.B., Im Y.H., Im S.A., Semiglazov V., Ciruelos E., Schneeweiss A., Loi S., Monturus E. (2020). Pertuzumab, trastuzumab, and docetaxel for HER2-positive metastatic breast cancer (CLEOPATRA): End-of-study results from a double-blind, randomised, placebo-controlled, phase 3 study. Lancet Oncol..

[12] Robert N.J., Goertz H.P., Chopra P., Jiao X., Yoo B., Patt D., Antao V. (2017). HER2-Positive Metastatic Breast Cancer Patients Receiving Pertuzumab in a Community Oncology Practice Setting: Treatment Patterns and Outcomes. Drugs Real World Outcomes.

[13] Vazquez J.C., Antolin S., Ruiz-Borrego M., Servitja S., Alba E., Barnadas A., Lluch A., Martin M., Rodriguez-Lescure A., Sola I. (2023). Dual neoadjuvant blockade plus chemotherapy versus monotherapy for the 74 treatment of women with non-metastatic HER2-positive breast cancer: A systematic review and meta-analysis. Clin. Transl. Oncol..

[14] Hurvitz S.A., Martin M., Symmans W.F., Jung K.H., Huang C.S., Thompson A.M., Harbeck N., Valero V., Stroyakovskiy D., Wildiers H. (2018). Neoadjuvant trastuzumab, pertuzumab, and chemotherapy versus trastuzumab emtansine plus pertuzumab in patients with HER2-positive breast cancer (KRISTINE): A randomised, open-label, multicentre, phase 3 trial. Lancet Oncol..

[15] Rimawi M., Ferrero J.M., de la Haba-Rodriguez J., Poole C., De Placido S., Osborne C.K., Hegg R., Easton V., Wohlfarth C., Arpino G. (2018). FirstLine Trastuzumab Plus an Aromatase Inhibitor, With or Without Pertuzumab, in Human Epidermal Growth Factor Receptor 2-Positive and Hormone ReceptorPositive Metastatic or Locally Advanced Breast Cancer (PERTAIN): A Randomized, Open-Label Phase II Trial. J. Clin. Oncol..

[16] Hurvitz S.A., Hegg R., Chung W.P., Im S.A., Jacot W., Ganju V., Chiu J.W.Y., Xu B., Hamilton E., Madhusudan S. (2023). Trastuzumab deruxtecan versus trastuzumab emtansine in patients with HER2-positive metastatic breast cancer: Updated results from DESTINY-75 Breast03, a randomised, open-label, phase 3 trial. Lancet.

[17] Xu B., Li W., Zhang Q., Li Q., Wang X., Li H., Sun T., Yin Y., Zheng H., Feng J. (2023). Pertuzumab, trastuzumab, and docetaxel for Chinese patients with previously untreated HER2-positive locally recurrent or metastatic breast cancer (PUFFIN): Final analysis of a phase III, randomized, double-blind, placebo-controlled study. Breast Cancer Res. Treat..

[18] Perez E.A., Barrios C., Eiermann W., Toi M., Im Y.H., Conte P., Martin M., Pienkowski T., Pivot X., Burris H. (2017). Trastuzumab Emtansine with or Without Pertuzumab Versus Trastuzumab Plus Taxane for Human Epidermal Growth Factor Receptor 2-Positive, Advanced Breast Cancer: Primary Results From the Phase III MARIANNE Study. J. Clin. Oncol..

[19] Shao Z., Tseng L.-M., Huang C.-S., Pang D., Yang Y., Li W., Liao N., Geng C., Zhang Q., Xu B. (2021). Pertuzumab and trastuzumab as adjuvant treatment for HER2-positive early breast cancer: Outcomes in Chinese patients in the APHINITY study. Jpn. J. Clin. Oncol..

[20] Ahn H.K., Sim S.H., Suh K.J., Kim M.H., Jeong J.H., Kim J.Y., Lee D.W., Ahn J.H., Chae H., Lee K.H. (2022). Response Rate and Safety of a Neoadjuvant Pertuzumab, Atezolizumab, Docetaxel, and Trastuzumab Regimen for Patients with ERBB2-Positive Stage II/III Breast Cancer: The Neo-PATH Phase 2 Nonrandomized Clinical Trial. JAMA Oncol..

[21] Shao Z., Pang D., Yang H., Li W., Wang S., Cui S., Liao N., Wang Y., Wang C., Chang Y.C. (2020). Efficacy, Safety, and Tolerability of Pertuzumab, Trastuzumab, and Docetaxel for Patients with Early or Locally Advanced ERBB2-Positive Breast Cancer in Asia: The PEONY Phase 3 Randomized Clinical Trial. JAMA Oncol..

[22] Miles D., Ciruelos E., Schneeweiss A., Puglisi F., Peretz-Yablonski T., Campone M., Bondarenko I., Nowecki Z., Errihani H., Paluch-Shimon S. (2021). Final results from the PERUSE study of first-line pertuzumab plus trastuzumab plus a taxane for HER2-positive locally recurrent or metastatic breast cancer, with a multivariable approach to guide prognostication. Ann. Oncol..

[23] Von Minckwitz G., Procter M., de Azambuja E., Zardavas D., Benyunes M., Viale G., Suter T., Arahmani A., Rouchet N., Clark E. (2017). Adjuvant Pertuzumab and Trastuzumab in Early HER2- Positive Breast Cancer. N. Engl. J. Med..

[24] Kuemmel S., Tondini C.A., Abraham J., Nowecki Z., Itrych B., Hitre E., Karaszewska B., Juárez-Ramiro A., Morales-Vásquez F., Pérez-García J.M. (2021). Subcutaneous trastuzumab with pertuzumab and docetaxel in HER2-positive metastatic breast cancer: Final analysis of MetaPHER, a phase IIIb single-arm safety study. Breast Cancer Res. Treat..

[25] Lee Y.P., Lee M.S., Kim H., Kim J.Y., Ahn J.S., Im Y.H., Park Y.H. (2022). Real-World Evidence of Trastuzumab, Pertuzumab, and Docetaxel Combination as a FirstLine Treatment for Korean Patients with HER2-Positive Metastatic Breast Cancer. Cancer Res. Treat..

